# Preoperative High-Dose Steroid Has Long-Term Beneficial Effects for Myasthenia Gravis

**DOI:** 10.1155/2013/709480

**Published:** 2013-07-14

**Authors:** Syuichi Tetsuka, Ken-ichi Fujimoto, Kunihiko Ikeguchi

**Affiliations:** Division of Neurology, Department of Internal Medicine, Jichi Medical University, 3311-1, Yakushiji, Shimotsuke, Tochigi 329-0498, Japan

## Abstract

Previous studies addressing preoperative steroid treatment have revealed that control of myasthenia gravis (MG) with steroids prior to surgery appeared to stabilize postoperative status. The purpose of our study was to clarify the clinical benefits of the preoperative programmed high-dose steroid treatment on the long-term outcomes of MG patients. We retrospectively reviewed the records of 171 MG patients who were followed up after undergoing thymectomy in our hospital between 1988 and 2006. One hundred and thirteen patients in the programmed treatment group had received preoperative steroid treatment, while 58 patients received no steroid treatment during the preoperative period. Clinical remission, which was defined as the achievement of the modified pharmacologic remission (PR) for at least 1 year, and clinical benefits were compared between the two groups. With regard to the remission after thymectomy, Kaplan-Meier life-table curves for patients in the preoperative steroid treatment group versus those for patients in the no steroid preoperative treatment group revealed a significantly higher probability of the PR in the preoperative steroid treatment group (log-rank test, *P* < 0.01). This study might be the first, as per our knowledge, to indicate that preoperative programmed high-dose steroid treatment has long-term beneficial effects for MG patients.

## 1. Introduction

The prognosis of myasthenia gravis (MG) has improved dramatically because of advances in critical care medicine and symptomatic treatments. The immunopathogenesis of MG is fundamentally a T-cell-dependent autoimmune process resulting from loss of tolerance toward self-antigens in the thymus. Thymectomy is based on this immunological background. MG patients that cannot be controlled with adequate symptomatic treatment or those who fail to achieve remission after thymectomy usually achieve remission after the addition of other immunotherapies. Steroids are the firstline of immunosuppressive treatment and the most consistently effective treatment for MG. Several studies of steroid treatment for MG found that remission or a marked improvement occurred in approximately 80% cases, and high-dose steroids are universally preferred for remission induction [1]. Immunosuppressive treatment comprising alternate-day administration of steroids before thymectomy was reported by Yamaguchi and associates to help stabilize the patient's condition after surgery [2]. MG patients have undergone thymectomy after programmed high-dose steroid treatment at our hospital since 1991. Endo et al. reported that programmed high-dose steroid treatment in MG patients is feasible and provides clinical benefits for preoperative fluctuating symptoms at our hospital [3]. Furthermore, preoperative steroid treatment seems to stabilize postoperative respiratory status without having adverse effects such as surgical infection in MG patients, and some studies have reported that thymectomy had no negative impact on morbidity and mortality in MG patients; in fact, the outcomes of patients taking steroids were better [3–6]. The primary benefit reported in these studies is stabilization of the postoperative status of MG patients, and no adverse effects of steroid therapy, such as surgical site infection or postoperative complications, were noted. Furthermore, steroid treatment before extended thymectomy is related to early palliation of MG symptoms. However, some researchers are of the opinion that steroid treatment should be avoided if possible because they increase the risk of side effects and that preoperative high-dose steroid treatment should be avoided considering the risk of initial deterioration. Furthermore, the long-term outcomes of this treatment remain unclear. Thus, in order to advocate this approach with increased confidence, it is necessary to investigate the effects of this treatment on long-term outcomes.

## 2. Patients and Methods

### 2.1. Patients Data

 We retrospectively evaluated the data of 171 patients who had undergone thymectomy for MG during an 18-year period (1988–2006, Table 1). As mentioned previous patients have undergone thymectomy after programmed high-dose steroid treatment at our hospital since 1991, and patients who did not received high-dose steroid treatment mainly were those before 1991. The diagnosis of MG was based on clinical and electromyographic evaluations and positive edrophonium or intramuscular neostigmine responses. Characteristic responses to low-frequency repetitive stimulation and antiacetylcholine receptor (AChR) antibody assays were supportive of the diagnoses. The indications for thymectomy included generalized nonthymomatous MG, ocular MG refractory to medical treatment, and evidence of thymoma. Preoperative disease severity was established according to the Osserman classification. Patients were divided into two groups on the basis of preoperative treatment: a programmed treatment group that received the programmed high-dose steroid treatment and a no preoperative steroid treatment group that was treated after surgery as required with different combinations of anticholinesterase agents and steroids. The preoperative programmed high-dose steroid treatment group comprised 113 patients, and the no preoperative steroid treatment group comprised 58 patients (Table 1). The study was conducted with the approval of the ethics committee at our institution.

### 2.2. Inductions for Preoperative Programmed High-Dose Steroid and Medication

 To decrease the risk of initial worsening of MG symptoms due to steroid administration, alternate-day oral administration of prednisolone (PSL) was initiated at 5–10 mg/day, which was then gradually increased by 5–10 mg/week up to 1.5 mg/kg (maximum 120 mg) [3, 7]. When patients achieved a PSL treatment with 1.5 mg/kg drug alternate day for 4–6 weeks, transsternal extended thymectomy was performed. A histamine H_2_ blocker, vitamin D, or bisphosphonate and potassium were routinely administered to all patients as prophylaxis for the adverse effects of steroids. Transsternal extended thymectomy was performed in both groups via median sternotomy under general anesthesia by selected skilled surgeons at our hospital [3].

### 2.3. Patient Followup and Outcome Evaluation

 After discharge from the hospital, patients visited the neurology outpatient clinic regularly every month, unless medications were discontinued. From 1 to 2 months postoperatively, reduction of PSL was started at the rate of 5 mg/month or less according to patient symptoms. Transition of PSL dose and representative side effects of steroid were recorded. Annual evaluation of medications and symptoms was conducted after thymectomy for both groups of patients. We defined that the end point of the study was the achievement of the modified pharmacologic remission (PR). In order to approach complete stable remission (CSR) as closely as possible, the modified PR was defined as the absence of symptoms and signs of MG for at least 1 year, with intake of a daily PSL dose of 5.0 mg or less for MG and no intake of other immunosuppressive drugs that we have set our own definition referring to the past clinical trials [8]. Prognostic factors including time of diagnosis from onset (<1 year), age at onset (<40 years), and severity of disease at diagnosis (Osserman classification 1, 2A) that predicted a better remission were analyzed separately in both groups [9]. The recurrence was defined as patients with the modified PR that subsequently developed clinical findings or increased more medication for MG.

### 2.4. Statistical Analysis

 For general statistical analyses, we used the SPSS v.11.0.1 program. Values for continuous data are presented as mean ± standard deviation and range. The *χ* ^2^ test for independent testing was used to compare categorical variables, and Student's *t*-test was applied to compare continuous variables. The Mann-Whitney's *U-*test was applied to the Osserman classification for independent testing between both groups. Kaplan-Meier life-table analysis was performed, and the log-rank test was used to evaluate effects of the variables examined on the distribution of the modified PR and the defined recurrence over time. The Cox proportional hazard model was applied to verify the concurrent effect of the evaluated factors on the modified PR. The following variables were included in the model: preoperative programmed high-dose steroid (yes versus no), sex (male versus female), age at onset (<40 years versus ≧40 years), thymoma (presence versus no), the Osserman classification (1, 2A versus 2B, 3, 4), and time of diagnosis from onset (<1 year versus ≧1 year). All tests were two-tailed, and significance was set at *P* < 0.05.

## 3. Results

### 3.1. Patients Characteristics

 Patient characteristics are listed according to group in Table 1. There was no significant difference in severity of disease, age of onset, sex, presence of thymoma, and time of diagnosis from onset between the two groups. And the number of MG patients who started to take additional immunosuppressive drugs after surgery was 10/113 (8.8%) in preoperative steroid treatment group and 1/58 (1.7%) in no preoperative steroid treatment group, with the difference not being significant (Table 1).

### 3.2. Postoperative Respiratory Failure and Myasthenic Crisis after Thymectomy

**Figure 1:**
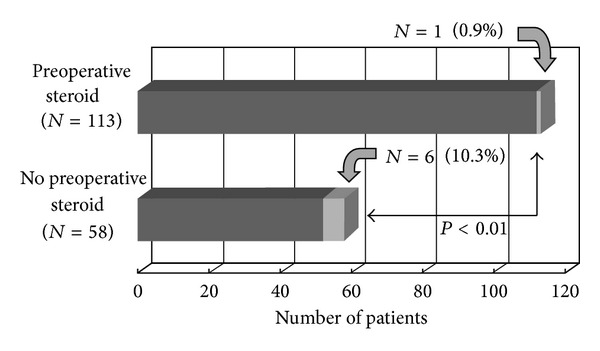
Postoperative respiratory failure and myasthenic crisis after thymectomy: postoperative respiratory failure and myasthenic crisis occurred only in a patient (0.9%) in preoperative steroid treatment group but did occur in 6 patients (10.3%) in no preoperative steroid treatment group during intraoperative period (*P* < 0.01, *χ* ^2^-test).

 Postoperative respiratory failure and myasthenic crisis occurred only in a patient (0.9%) in preoperative steroid treatment group but did occur in 6 patients (10.3%) in no preoperative steroid treatment group during intraoperative period (*P* < 0.01, *χ* ^2^-test, Figure 1). Preoperative high-dose steroid treatment was found to be the largest impact as well as previous studies.

### 3.3. Probability of the Modified PR in Both Groups

**Figure 2:**
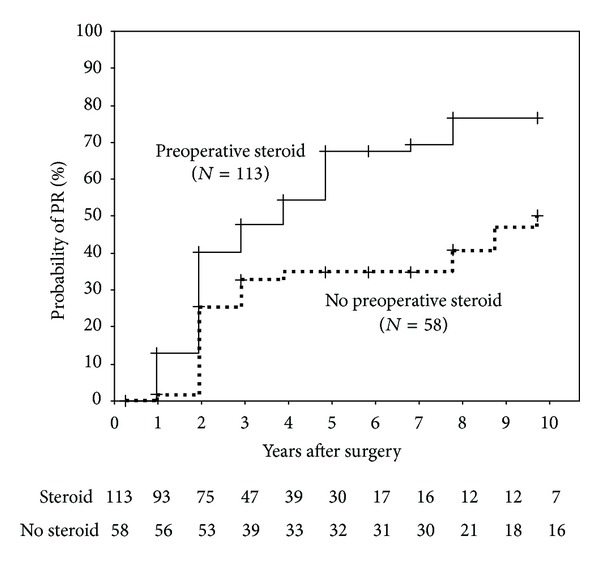
Kaplan-Meier curves for the modified PR in MG patients: Kaplan-Meier curves for MG patients (*N* = 113) in preoperative programmed high-dose steroid treatment group (solid line) versus those (*N* = 58) in no preoperative steroid treatment group (broken line). The modified PR was defined as the absence of symptoms and signs of MG for at least 1 year, with intake of a daily PSL dose of 5.0 mg or less for MG and no intake of other immunosuppressive drugs. Vertical bars indicate censured data. A significant difference was found between the two curves (log-rank test, *P* < 0.01). Therefore the present study aimed to clarify the clinical benefits of preoperative programmed high-dose steroid treatment for MG by comparing the long-term outcomes of MG patients who received high-dose steroid treatment prior to thymectomy with those of MG patients who did not.

 According to the Kaplan-Meier life table analysis, the probability of the modified PR in the preoperative steroid treatment group was 40%, 54%, and 76.5% at 3, 5, and 10 years, respectively. On the other hand, the probability of the modified PR in the no preoperative steroid treatment group was 32.5%, 34.5%, and 49% at 3, 5, and 10 years, respectively (Figure 2). With regard to the modified PR after thymectomy, Kaplan-Meier life-table curves for patients in the preoperative programmed high-dose steroid treatment group versus those for patients in the no preoperative steroid treatment group revealed a significantly higher probability of the modified PR in the former group (log-rank test, *P* < 0.01, Figure 2). 

In the multivariate analysis accounting for investigated variables, significant effects were found for no preoperative high-dose steroid treatment (hazard ratio, 1.946, 95% confidence interval, 1.188–3.189, *P* = 0.008), presence of thymoma (hazard ratio, 1.901, 95% confidence interval, 1.025–3.526, *P* = 0.041), and time of diagnosis from onset (≧1 year) (hazard ratio, 1.660, 95% confidence interval, 1.026–2.686, *P* = 0.039) as prognostic factors in MG patients after thymectomy(Table 2). Thus, preoperative programmed high-dose steroid treatment was an independent predictor of the modified PR in the multivariate analysis. The other values were not significant (Table 2).

### 3.4. Probability of Recurrence after the Modified PR in Both Groups

**Figure 3:**
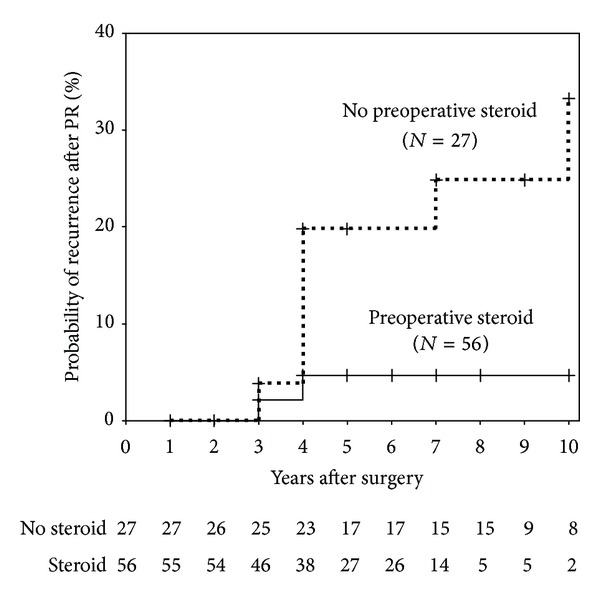
Kaplan-Meier curves for the defined recurrence after the modified PR status: there were 83 MG patients that achieved the modified PR status during followup (preoperative steroid, 56, no preoperative steroid, 27) without censure. These MG patients in the no preoperative steroid treatment group (broken line) exhibited a significantly higher rate of the defined recurrence than those in the preoperative steroid treatment group (solid line) (log-rank test, *P* = 0.03). Vertical bars indicate censured data.

 Kaplan-Meier curves analysis for the defined recurrence of MG patients after the modified PR status was performed. There were 83 MG patients that achieved the modified PR status during followup (preoperative steroid; 56, no preoperative steroid, 27) without censure. In contrast, these MG patients in the no preoperative steroid treatment group exhibited a significantly higher rate of the defined recurrence than those in the preoperative steroid treatment group (log-rank test, *P* = 0.03, Figure 3). 

### 3.5. Cumulative Frequency of Steroid Treatment after Thymectomy in the No Preoperative Steroid Group

**Figure 4:**
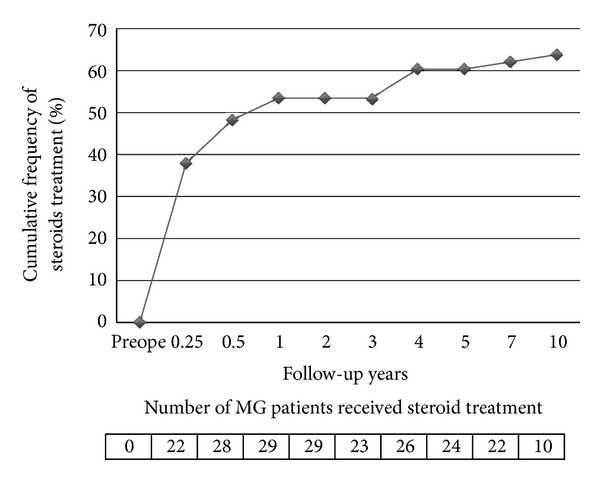
We show cumulative frequency of steroid treatment in the no preoperative steroid group after thymectomy during follow-up period. The life table analysis of steroid treatment started at zero. And we show the number of MG patients who received steroid treatment, at which time in the course of their diseases at the bottom.

 We show the cumulative frequency of steroid treatment after thymectomy in the no preoperative steroid group during follow-up period (Figure 4). The life table analysis of steroid treatment started at zero. The cumulative frequency increased gradually, and 10 years after thymectomy, it increased to 64% in the no preoperative steroid group.

### 3.6. Steroid Side Effects after Thymectomy in the Preoperative Steroid Group

Steroid side effects after thymectomy during follow-up period in the preoperative programmed high-dose steroid treatment group are listed in Table 3. Side effects were documented in 42/133 patients (37.2%) during follow-up period, with hyperglycemia and diabetes mellitus being the most common.

### 3.7. Probability of the Modified PR with or without Thymoma

**Figure 5:**
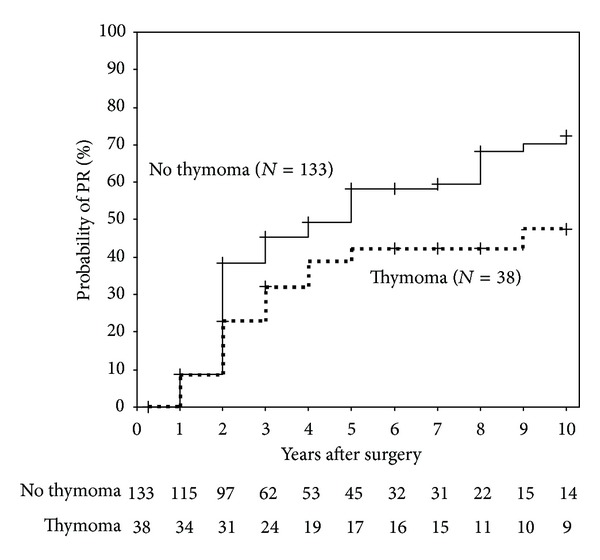
The modified PR is shown in thymoma patients (broken line) compared with nonthymoma patients (solid line). Vertical bars indicate censured data. A significant difference was found between the two curves (log-rank test, *P* = 0.04).

 Furthermore, when patients either with or without thymoma were compared, Kaplan-Meier life-table analysis revealed a significantly higher probability of the modified PR in the absence of thymoma (log-rank test, *P* = 0.04, Figure 5).

## 4. Discussion

It should be noted that there is currently no clear evidence regarding the optimal timeframe for steroid dose reduction, the speed with which steroids can be safely reduced, and duration of treatment [10]. Therefore, it was difficult to define CSR as the endpoint in our study. Thus, we have set our own definition of PR as the endpoint referring to the past clinical trials [7]. By using the end point of the modified PR, this study revealed remarkable long-term beneficial effects of preoperative programmed high-dose steroid treatment on the modified PR in MG patients. The following prognostic factors for MG are currently regarded as important for predicting remission: time of diagnosis from onset (<1 year), age at onset (<40 years), severity of disease at onset, sex, and presence of thymoma [9, 11, 12]. Our study revealed the probability of the modified PR in the programmed high-dose steroid treatment group was significantly higher than that in the no preoperative steroid treatment group, and this finding was independent of the previously mentioned prognostic factors in the multivariate analysis. Furthermore, it is important to know whether, during followup, MG patients could keep the modified PR status without disease deterioration to indicate the long-term beneficial effects of preoperative programmed high-dose steroid treatment. In our study, we could indicate that MG patients in the no preoperative steroid treatment group exhibited a significantly higher rate of the defined recurrence than those in the preoperative steroid treatment group (Figure 3). 

Although MG patients in the no preoperative steroid treatment group did not receive steroids before thymectomy, many of them were gradually required to use steroids for disease control after thymectomy (Figure 4). These observations suggest that the timing of commencing steroid use is not critical. The therapeutic effect of thymectomy generally occurs after approximately 1 year, and remission is most likely in the 5–10-year period following surgery [13, 14]. The reported CSR after thymectomy has varied from 8.3% to 53.1% in previous studies [14, 15]. The underlying reason is that anti-AChR antibodies are produced not only in the thymus but also in external tissues, including peripheral lymph nodes and bone marrow. Therefore, the additional immunotherapies such as those with steroids are anyways required in many MG patients after thymectomy. The same fact was recognized even in our study.

A limitation of this study was that we compared a little different time periods of the two groups. Because results of treatment generally improve with time by various causes, this factor might influence results of our study. However, significant effect of preoperative programmed high-dose steroid treatment was remarkable high as an independent predictor of the modified PR in MG patients after thymectomy in the multivariate analysis (Cox regression analysis, *P* = 0.008). Thus, taking this thing together with our other previously mentioned data and previous reports, we considered that the result had sufficient reliability.

The autoimmune nature of the disease underlies the rationale for using steroids, considering that the beneficial effects of this group of drugs have been observed in other autoimmune diseases. The mechanisms of action of steroids in MG patients are poorly understood. Effects on the activation of helper T cells and the proliferation of B cells, activated T cells, and antigen-presenting cells are considered to play a role [16]. We suggest that one of the reasons for the clinical effects of preoperative high-dose steroid treatment is the blockage of pathways by which immunoactivation causes cellular damage and undesirable clinical outcomes at a stretch. Moreover, the high doses generally used to induce remission can also induce apoptosis of various immune cells [17, 18].

PSL was used in the majority of MG patients. Although we were able to reveal that preoperative programmed high-dose steroid treatment has long-term beneficial clinical effects in MG patients, a critical factor in the treatment of MG patients is the occurrence of steroid-induced side effects. Numerous steroid side effects of varying severity were reported, including osteoporosis, diabetes mellitus, infection, gastric ulcer, and glaucoma among others, and at least one side effect was observed in 52.2% patients of MG patients [1]. But short courses of large intravenous doses were reportedly employed to manage exacerbations with good results [19]. Sanders and Evoli reported that at least 30% of their patients developed some side effects, usually weight gain and easy bruising, until the dose was decreased to 20 mg on alternate days or less [20]. Steroid side effects were also observed to some degree in our study, with hyperglycemia and diabetes mellitus being the most common. Diabetes is a strong but not absolute contraindication to steroids. Glucose control requires adjustment of hypoglycemic agents, and it can be especially challenging if an alternate-day regimen is attempted. In such patients, we may administer a daily dose while decreasing the amount as rapidly and as completely as the disease allows. The many severe side effects of long-term steroid use indicate that such a long-term use should be avoided. Therefore, other immunosuppressive drugs could be coadministered to enable a decrease in the steroid dosage [21–23], although these agents may have their own side effects that require monitoring [13, 24].

Thymoma has been reported as a factor associated with worse neurological outcome and symptom relapse after thymectomy [25, 26]. The results from this study showed that thymomatous MG was a risk factor for the modified PR. Although the mechanisms underlying the effects of thymoma on MG remain unclear, several possibilities exist. For example, thymoma is more common among older patients so that MG may be relatively more severe in patients with thymoma [11]. Alternatively, patients with thymoma may have an increased tendency for associated autoimmune disorders and extrathymic neoplasms [27]. It is also possible that residual thymoma or thymus after thymectomy may influence the outcome of MG treatment [28].

## 5. Conclusion

In conclusion, previous studies have revealed that preoperative programmed high-dose steroid treatment for the control of MG before surgery apparently stabilizes postoperative status. Ours might be the first study, as per our knowledge, to indicate that preoperative programmed high-dose steroid treatment has long-term beneficial effects for MG patients.

## Figures and Tables

**Table 1:** Patients characteristics.

Characteristics	Preoperative high-dose steroid (*N* = 113)	No preoperative steroid (*N* = 58)	*P* value
Male/female (sex)	38/75	27/31	0.10 (*χ* ^2^-test)
Onset age years	45.8 (±18.9)	41.3 (±18.2)	0.14 (*t-*test)
<40/≧40 (onset age years)	43/70	29/29	0.13 (*χ* ^2^-test)
Thymoma (±)	22/91	16/42	0.23 (*χ* ^2^-test)
<1/≧1 year (time of diagnosis from onset)	70/43	30/28	0.20 (*χ* ^2^-test)
Osserman			
1	22	9	
2A	39	14	0.19 (*U-*test)
2B	40	28	
3	9	4	
4	3	3	
(1, 2A/others)	61/52	23/35	0.08 (*χ* ^2^-test)
Immunosuppressant drug use after surgery (±)	10/103	1/57	0.07 (*χ* ^2^-test)
Follow-up duration (years)	5.3 (±3.4)	6.9 (±2.6)	<0.05 (*U-*test)

Data were expressed by mean (Standard deviation: SD).

**Table 2:** Cox regression analysis of prognostic factors in MG patients after thymectomy.

Factors	Hazard ratio	95% CI	*P* value
No preoperative high-dose steroid	1.946	(1.188–3.189)	0.008*
Sex (male)	0.936	(0.585–1.496)	0.781
Age at onset (≧40 years)	1.014	(0.630–1.631)	0.954
Thymoma (+)	1.901	(1.025–3.526)	0.041*
Osserman (2B, 3, 4)	0.880	(0.559–1.386)	0.582
Time of diagnosis from onset (≧1 year)	1.660	(1.026–2.686)	0.039*

*Significant covariates, CI: confidence interval, and Hazard ratios refer to the categories interval.

**Table 3:** Steroid side effects in the preoperative programmed high-dose steroid treatment group.

Steroid side effects	Frequency
Total	42/113 (37.2%)
Hyperglycemia, diabetes mellitus	22/113 (19.5%) (Insulin treatment, 6)
Infection	12/133 (10.6%) (Herpes zoster, 4, candida esophagitis, 2, Septicemia, 2, pneumonitis, 1, pleuritis, 1, phlegmon, 1, mediastinitis, 1)
Gastric ulcer	3/113 (2.7%)
Anxiety/depression (steroid psychosis)	3/113 (2.7%)
Bone fracture	1/113 (0.9%)
Delay of wound recovery	1/113 (0.9%)
